# Long-Term Hypoxia Maintains a State of Dedifferentiation and Enhanced Stemness in Fetal Cardiovascular Progenitor Cells

**DOI:** 10.3390/ijms22179382

**Published:** 2021-08-29

**Authors:** Cole Knox, Victor Camberos, Lourdes Ceja, Andrea Monteon, Lorelei Hughes, Lawrence Longo, Mary Kearns-Jonker

**Affiliations:** 1 Department of Pathology and Human Anatomy, Loma Linda University School of Medicine, Loma Linda, CA 92350, USA; cknox@students.llu.edu (C.K.); vcamberos@students.llu.edu (V.C.); lceja@llu.edu (L.C.); amonteon@llu.edu (A.M.); loreleihughes@students.llu.edu (L.H.); 2 Lawrence D. Longo MD Center for Perinatal Biology, Loma Linda University School of Medicine, Loma Linda, CA 92350, USA; llongo@llu.edu

**Keywords:** hypoxia, stemness, cardiovascular progenitor cells, Islet-1, ovine

## Abstract

Early-stage mammalian embryos survive within a low oxygen tension environment and develop into fully functional, healthy organisms despite this hypoxic stress. This suggests that hypoxia plays a regulative role in fetal development that influences cell mobilization, differentiation, proliferation, and survival. The long-term hypoxic environment is sustained throughout gestation. Elucidation of the mechanisms by which cardiovascular stem cells survive and thrive under hypoxic conditions would benefit cell-based therapies where stem cell survival is limited in the hypoxic environment of the infarcted heart. The current study addressed the impact of long-term hypoxia on fetal Islet-1+ cardiovascular progenitor cell clones, which were isolated from sheep housed at high altitude. The cells were then cultured in vitro in 1% oxygen and compared with control Islet-1+ cardiovascular progenitor cells maintained at 21% oxygen. RT-PCR, western blotting, flow cytometry, and migration assays evaluated adaptation to long term hypoxia in terms of survival, proliferation, and signaling. Non-canonical *Wnt*, *Notch*, *AKT*, *HIF*-*2α* and *Yap1* transcripts were induced by hypoxia. The hypoxic niche environment regulates these signaling pathways to sustain the dedifferentiation and survival of fetal cardiovascular progenitor cells.

## 1. Introduction

Cardiovascular progenitor cells (CPCs) have been evaluated for numerous applications to treat a variety of cardiovascular diseases from congenital heart defects to myocardial infarctions [1,2]. This population of cells is capable of differentiating into the three cardiac lineages: cardiomyocytes, endothelial, and vascular smooth muscle cells. In addition to their multipotent capacity, these cells can be harvested on a patient-specific basis and rapidly expanded to obtain adequate cell numbers for in vitro experimentation and therapeutic applications [3,4]. To complement these intrinsic qualities in therapeutic applications, it is essential that we understand how to program these cells during and throughout both culture and clinical application. The field of stem cell programming continues to develop to meet these needs.

The microenvironment of a cell includes not only chemical differentiation signals but also physical differentiation factors such as sheer force, osmolarity, pressure, stretch, and oxygen tension. These physical factors remain a popular topic for experimentation, as each factor plays an individual role in controlling differentiation. An optimal method of controlled differentiation is yet to be established; however, recent findings suggest that oxygen tension regulates differentiation and migratory capacity during fetal development [5]. 

Typically, fetal CPCs are cultured under normoxic (21% oxygen) conditions supplemented by 5% CO_2_; however, naturally, these cells develop within low oxygen tension environments around 1% oxygen. When the maternal decidua arterializes, oxygen tensions increase to ~5–7%, initiating fetal progenitor cell migration and differentiation. This phenomenon leads us to hypothesize that oxygen tension plays a regulative role in maintaining fetal cardiovascular progenitor cell survival, migration, and pluripotency [6,7,8,9,10]. 

Previously, several labs, including our own, have explored preconditioning cells with short-term hypoxia exposure to evaluate CPC function prior to transplantation. Short-term hypoxic preconditioning enhanced both pro-survival signaling and invasion ability prior to therapeutic application [3,11]. Enhanced stem cell properties observed in these cells are the result of AKT phosphorylation induced by short-term hypoxia [3]. The effects of long-term hypoxia exposure on fetal Islet-1+ CPCs has not been evaluated, to our knowledge. 

The neuregulin/ERBB signaling pathway plays an essential role in cardiac trabeculation, proliferation, and in preventing apoptosis when overexpressed [12]. These downstream effects arise from the ERBB induction of PIK3C2B, which asserts its actions through AKT and MAPK [13]. The wingless tyrosine kinase (Wnt) pathways (non-canonical and canonical) promote cell fate specification, proliferation, and migration through NFk-B, which connects Wnt and AKT pathway signaling [14,15,16]. FAK induces AKT and plays a role in the regulation of apoptosis, proliferation, transcription, and migration within the developmental niche [17,18,19]. Notch functions to regulate differentiation, proliferation, and cell organization gestationally [20,21]. It has also been shown to maintain a propagative state in embryonic stem cells without spontaneous differentiation [22]. Notch induces AKT expression through the upregulation of Hes1 [23]. The Hippo pathway is well established as a key regulator of organ size and development. Specifically, Yap1 expression and translocation into the nucleus are associated with promoting a proliferative, anti-apoptotic cell state [24,25,26,27,28]. The nuclear translocation of Yap1 requires PI3K/AKT pathway activation in order to elicit its survival mechanisms [29,30]. Lastly, hypoxia inducible factors, HIF-1α and HIF-2α, have also been evaluated to determine their involvement in dedifferentiation and survival. 

These pathways not only regulate survival, proliferation, migration, and differentiation but also play roles in fetal development, which ties them to hypoxic regulation. In this study, we demonstrate the involvement of these pathways in the hypoxic niche and suggest a potential interconnected pathway that maintains the survival of dedifferentiated cardiovascular progenitor cells through the upregulation of the PI3K/AKT pathway by Notch, non-canonical Wnt, and FAK pathways.

## 2. Results

### 2.1. Cardiovascular Progenitor Cell Characterization

**Figure 1:**
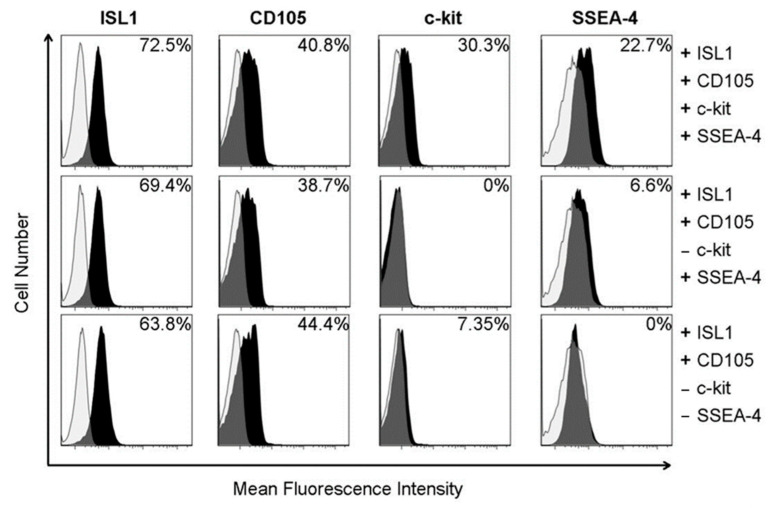
Fetal cardiovascular progenitor cells were isolated, monoclonally expanded, and evaluated using flow cytometry to analyze the expression of cardiovascular progenitor cell markers and the phenotypic population prevalence. A shift in the dark peak demonstrates the positive presence of each indicated protein. The percentages in the top right of each graph indicate the cell count percentage positive for each protein.

Fetal CPCs from both normoxic and hypoxic sheep were harvested for this study. Monoclonal cell populations were expanded and grouped based on their expression of Islet-1, c-KIT, CD105, and SSEA-4 via flow cytometry (Figure 1). Islet-1 is a known marker of early lineage, multipotent CPCs. The expression of SSEA-4 is representative of the invasiveness of the cells in this population. The cardiovascular progenitor clones chosen for this study were selected based on the triple positive expression of Islet-1, c-kit, and SSEA-4, as stemness and invasiveness enhance therapeutic potential.

### 2.2. Wnt, Notch, and FAK Function Synergistically to Promote Cell Survival

**Figure 2:**
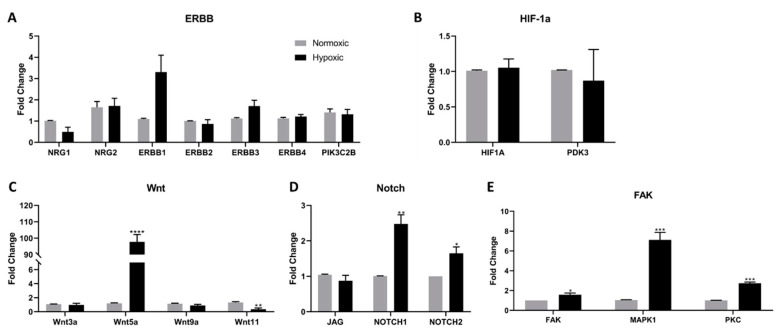
Real-time polymerase chain reaction quantification evaluating the regulation of five separate pathways including key molecules and downstream effectors: (**A**) NRG/ERB-B, (**B**) HIF-1a, (**C**) Wnt, and (**D**) Notch (**E**) FAK. Data are reported as the mean ± SEM, * *p* < 0.05, ** *p* < 0.01, *** *p* < 0.001, **** *p* < 0.0001.

Pathways that have been previously connected to hypoxic regulation in other models were evaluated in Islet-1+ early CPC clones following long term hypoxia exposure. These pathways include NRG/ERBB, HIF-1α, non-canonical Wnt, Notch, and FAK. Using RT-PCR, we found that *NRG* and *ERBB* expression remained unchanged, suggesting that this pathway does not play a role in long-term hypoxic regulation (Figure 2A). Neither *HIF*-*1α*, which has been demonstrated to increase in short-term hypoxia [3,11], or its downstream regulator *PDK3* were affected by long-term hypoxia (Figure 2B). The non-canonical Wnt pathway has an active role in promoting cell survival and preventing apoptosis. In our hypoxia model, non-canonical *Wnt5a* expression increased 98-fold, while concurrently, the expression of *Wnt11*, a member of the canonical Wnt pathway, was inhibited 0.36-fold (Figure 2C). Canonical Wnt signaling downregulation attenuates cell fate specification. Increased Notch signaling fosters cellular growth and survival [20,21]. Here, despite low oxygen tensions, *Notch* signaling was induced, suggesting enhanced survival in cardiovascular progenitor cells cultured in hypoxic conditions (Figure 2D). The expression of *FAK*, *MAPK1*, and *PKC* transcripts was upregulated following hypoxic conditioning (Figure 2E). Interestingly, these genes, as well as *NOTCH1*, promote AKT signaling activation. The upregulation of the *PI3K*/*AKT* pathway could also explain the lack of HIF-1α induction, as AKT is known to counter these effects in cardiomyoblast populations [29].

### 2.3. FAK and Notch Directly Upregulate PI3K/AKT in the Hypoxic Niche

**Figure 3:**
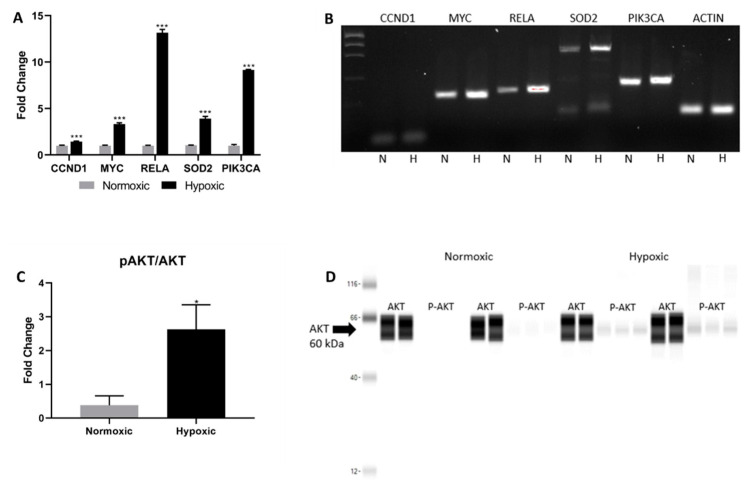
(**A**) Real time polymerase chain reaction data evaluating AKT pathway activity. (**B**) Agarose gel confirmation of rt-PCR products. (**C**) ProteinSimple analysis showing the phosphorylated AKT/AKT ratio. (**D**) The corresponding ProteinSimple automated Western blot quantifying AKT expression. Data are reported as the mean ± SEM, * *p* < 0.05, *** *p* < 0.001.

AKT signaling activation is associated with enhanced proliferation and survival. Notch regulation of AKT signaling stems from the induction of Hes1, which directly activates AKT and its downstream effectors [21,23]. Similarly, the increased expression of FAK, MAPK, and PKC observed in the hypoxic niche promote AKT-dependent survival and resistance to oxidative stress [19]. To further elucidate the mechanism by which the AKT signaling pathway is induced, we confirmed the upregulation of several additional transcripts in the AKT signaling pathway via RT-PCR. For example, *CCND1* and *SOD2* were upregulated in CPCs cultured in hypoxia (Figure 3A,B). These genes are associated with increased cell cycle activity and the protection against reactive oxygen species released during oxidative stress, respectively [18,19]. Next, we assessed AKT phosphorylation via Western blot (Figure 3C,D). The hypoxic niche increased the ratio of phosphorylated AKT relative to its non-phosphorylated counterpart, confirming an activation of AKT signaling.

**Figure 4:**
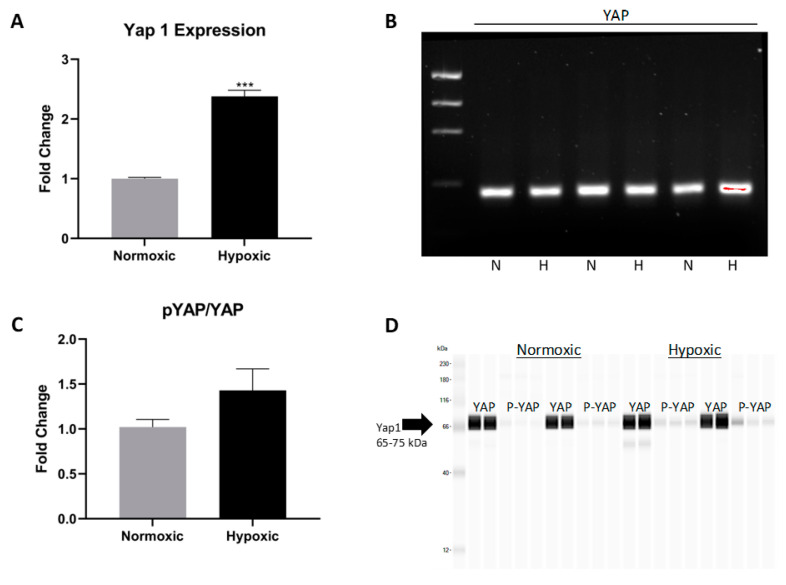
(**A**) ProteinSimple automated Western blot quantifying Yap1 expression. (**B**) 2% agarose gel confirming Yap1 amplification across normoxic and hypoxic cell lines. (**C**,**D**) ProteinSimple analysis showing the phosphorylated YAP/YAP ratio and the associated Western blot. Data are reported as the mean ± SEM, ** *p* < 0.01.

The AKT pathway promotes survival through many downstream effectors including Yap1, a member of the Hippo pathway. The phosphorylation of Yap1 results in cytoplasmic retention and eventually apoptosis or degradation. The nuclear translocation of non-phosphorylated Yap1 allows downstream targets to fulfill their role of driving cell proliferation and enhancing cell survival [30]. In response to hypoxia, CPCs presented with an increase in *Yap1* gene expression (Figure 4A,B). While an increase of *Yap1* expression was observed via RT-PCR, phosphorylated Yap1 was statistically unchanged, according to Western blotting, contrary to what we hypothesized (Figure 4C,D). In long-term hypoxia, the levels of phosphorylated/inactive Yap1 are not significantly elevated, suggesting that active Yap1 continues to enter the nucleus and promote cell survival.

### 2.4. Islet-1+ Cardiovascular Progenitor Cells Remain Dedifferentiated under Long-Term Hypoxic Conditions

**Figure 5:**
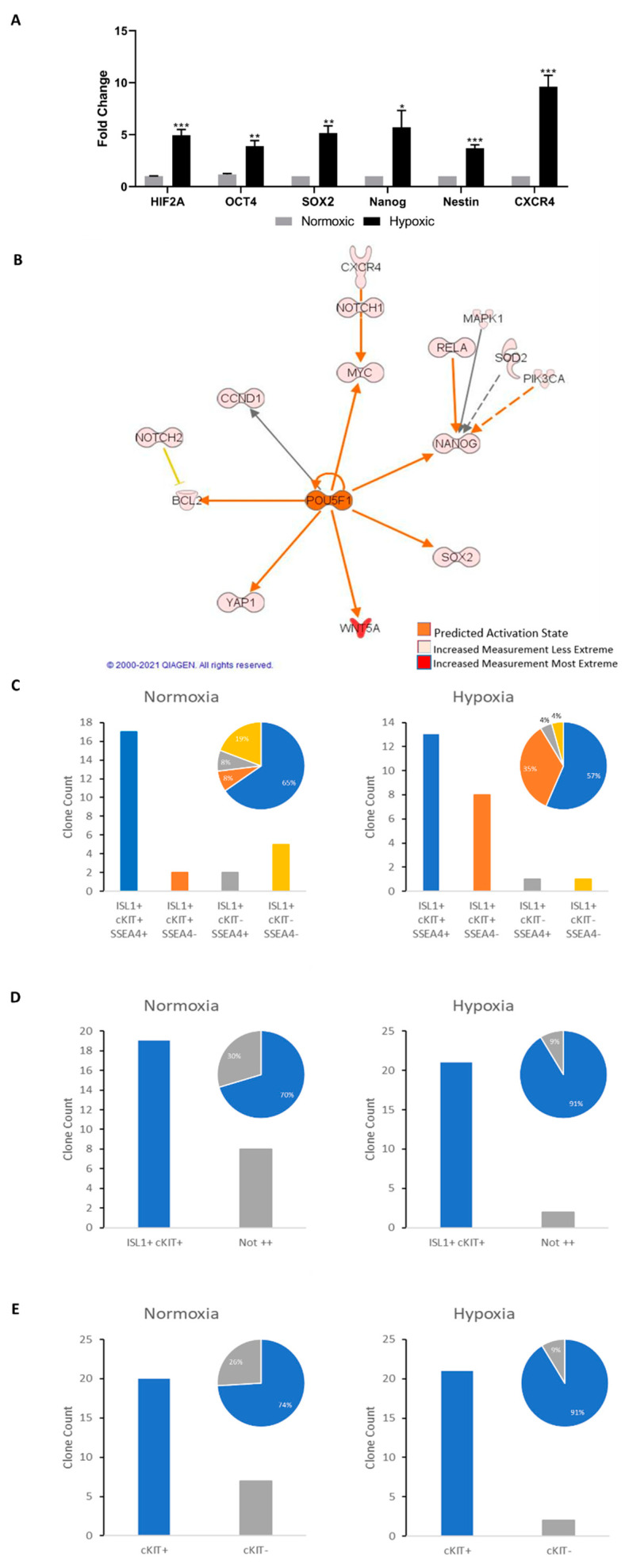
(**A**) PCR quantification representing the hypoxia inducible factor pathway fold changes of hypoxic cells compared to the normoxic controls. (**B**) IPA prediction of dedifferentiation pathway activity. POU5F1 exists as an alternative name for OCT4. (**C**–**E**) Quantification of flow cytometry data showing cell count and pie chart percentages of the representative isolated cell clones. Data are reported as the mean ± SEM, * *p* < 0.05, ** *p* < 0.01, *** *p* < 0.001.

HIF-2α is a known regulator of OCT4, SOX2, and Nanog expression in embryonic stem cells subjected to hypoxia through the nuclear translocation and activation of hypoxic response elements [6,31,32]. The long-term cell culture of CPCs corroborated this data, as verified by PCR analysis and predicted by Ingenuity Pathway Analysis (IPA) (Figure 5A,B and Supplemental Figure S1). *HIF*-*2α*, *OCT4*, *SOX2*, and *Nanog* were upregulated in hypoxic Islet-1+ cell clones and indicate that long-term hypoxia induces the dedifferentiation of fetal CPCs. Nanog transcript levels were found to decline following subsequent exposure to normoxic conditions (Supplemental Figure S2). *Nestin* is a known stemness marker in progenitor cells [33] and was elevated under hypoxic conditions. Similarly, *CXCR4* was upregulated in hypoxic conditioning, suggesting increased stemness [34]. Long-term hypoxia induced c-Kit expression and maintained Islet-1 expression, as shown by flow cytometry (Figure 5C–E). SSEA-4, typically known as a marker of invasiveness [35], was not induced, suggesting that fetal cells in a hypoxic niche stay harbored in said niche.

### 2.5. Cell Cycle Progression and Migration Are Unaffected by Long Term Hypoxia

**Figure 6:**
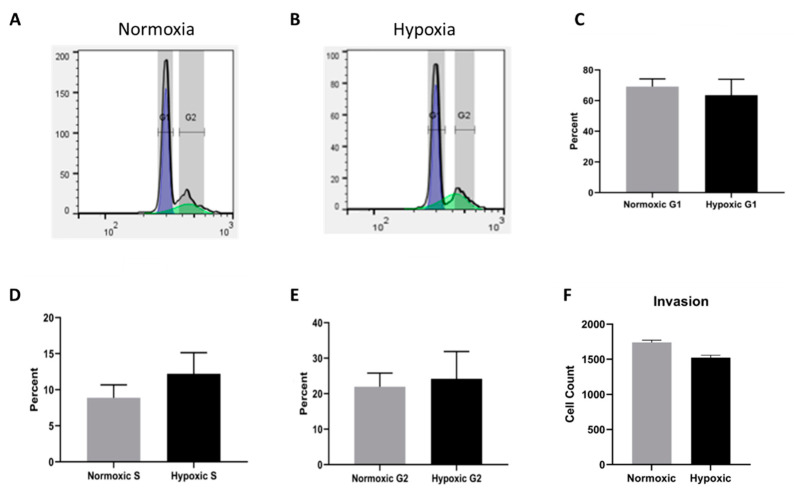
(**A**,**B**) Representative flow cytometry tracings of normoxic and hypoxic CPC clones for cell cycle analysis. (**C**–**E**) Quantification of the percentages of cells in G1, S, and G2 using the Dean-Jett-Fox model of cell cycle analysis. (**F**) Quantification of the migration data showing the cell count that successfully migrated.

To assess whether cell cycle progression is impacted by long-term hypoxic conditions, we conducted cell cycle analysis by flow cytometry. A representative tracing of both normoxic and hypoxic populations can be seen in Figure 6A,B. The data were quantified using a Dean-Jett-Fox model. As shown in Figure 6C–E, no significant difference was identified when comparing cell cycle progression in normoxic and hypoxic CPCs. Changes in oxygen tension similarly had no effect on invasion, suggesting that the upregulated signaling pathways did not affect the invasive capacity of fetal CPCs.

## 3. Discussion

Short-term hypoxic culture beneficially impacts CPCs such that survival and invasiveness are enhanced via AKT activation and SDF-1α sensitization [3]. Hypoxia inducible factors appear to be at the forefront of short-term hypoxic survival, as they interact with hypoxia response elements on the DNA and upregulate transcripts associated with survival [11,32]. We report here that the long-term maintenance of CPCs under hypoxic conditions promotes stemness and a state of dedifferentiation. These adaptations are the direct result of the upregulation of several interconnected signaling pathways. The long-term hypoxic response and the mechanisms associated with cell survival differ from the response to short-term hypoxia in early-stage CPCs. According to our research, there exists a common theme of AKT upregulation that connects both short- and long-term hypoxia, but it seems that differences arise when evaluating hypoxia inducible factors. Short term hypoxia results in the upregulation of *HIF*-*1* and downstream survival molecules such as *BCL2* and *HMOX* [3]. This current research suggests that long term hypoxic culture deals with survival through differing pathways including non-canonical *Wnt*, *FAK*, *Notch*, and *Yap*. *HIF*-*2α* and its downstream dedifferentiation factors are unique to long-term hypoxia and may need additional time and stress to activate dedifferentiation. *OCT4*, *SOX2*, and *Nanog* transcripts are not elevated in short-term hypoxic CPCs [3,36].

The hypoxic fetal microenvironment, as well as long-term culture under hypoxic conditions *in vitro,* regulates survival and dedifferentiation pathways. Non-canonical Wnt, Notch, FAK, AKT, and Yap signaling contributes to cell survival despite incredibly low oxygen tensions. The pathways discussed here interact to assert an anti-apoptotic state, reduce oxidative stress, and promote viability despite the altered nutrient intake of the cell in the hypoxic niche. Often, these pathways are uncontrollably upregulated in the cancer microenvironment, leading to unfettered proliferation, anti-apoptosis, and survival [10,37]. In contrast, the long-term hypoxic developmental niche maintains a level of control despite the low oxygen tension stressor of the microenvironment. 

*HIF*-*2α* remains upregulated, leading to the increased expression of hypoxic response elements and the transcription of genes responsible for SOX2, OCT4, and Nanog. The reexposure of hypoxic cells to a normoxic niche downregulated Nanog expression, confirming the level of control hypoxia has over dedifferentiation. These adaptations suggest that hypoxia promotes dedifferentiation in fetal ovine CPCs [6]. Dedifferentation could potentially broaden the multipotency of fetal CPCs, thereby widening their potential for therapeutic applications. The exact lineage staging must be further evaluated in order to fully understand the possibilities of long-term hypoxia in stem cell reprogramming.

**Figure 7:**
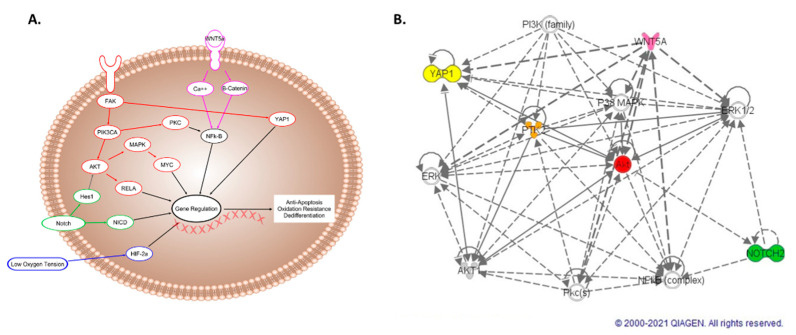
(**A**) Proposed interconnected pathway that regulates the dedifferentiated survival state found in hypoxic fetal cardiovascular progenitor cells. Each individual pathway is color-coordinated: AKT—Red, Notch—Green, HIF-2α—Blue, Wnt—Pink. (**B**) Dashed lines represent indirect interactions and solid lines represent direct interactions in the interactive network generated using IPA software (Qiagen).

We have elucidated five different pathways that contribute to sustaining a dedifferentiated survival state in fetal cardiovascular progenitor cells when subjected to a long-term hypoxic niche. In Figure 7, we propose and support a mechanism by which these pathways interconnect with a network identified using IPA [38,39,40,41,42,43,44,45,46,47,48,49,50,51,52,53,54,55,56,57,58,59,60,61,62,63,64,65,66,67,68,69,70,71,72,73,74,75]. These interactions influence the endpoints of hypoxic regulation and are connected either directly or indirectly acting through intermediates such as Hes1, FAK, and NFk-B [50,61,69,73]. Notch and AKT both interact with Hes1 to activate genes that regulate antiapoptosis, oxidative resistance, and dedifferentiation [73,74,75]. Yap1 and FAK influence AKT, achieving the same goals [40]. NFk-B interacts with non-canonical Wnt, Notch and AKT [61,69,73] Understanding the role of individual signaling pathways that function together in maintaining a dedifferentiated survival state provides insight into cellular adaptation to hypoxia. This information is relevant both in organismal development and as a potential pretreatment for therapeutic application.

Cardiovascular progenitors are currently being evaluated for their potential use in novel therapeutic applications following physical differentiation protocols involving sheer force, transmural pressure, and oxygen tension [3,4]. These physical differentiation factors can potentially introduce unwanted differentiation in multipotent stem cells, hindering their efficacy for regenerative applications. Long-term hypoxia could potentially augment therapies at the pre-treatment stage and broaden the therapeutic potential of progenitor cells by maintaining multipotency. The new information provided in this study addresses the impact of the microenvironment on differentiation while contributing to our understanding of stem cell potential for therapeutic applications. 

## 4. Materials and Methods

### 4.1. Animal Selection

The high altitude long-term hypoxic sheep were housed at Barcroft Laboratory, White Mountain Research Station, Bishop, CA, USA (altitude 3820 m; PaO_2_ 60 ± 2 mmHg) beginning at 30 days gestation. The pregnant females were transported from altitude prior to delivery and subsequently sacrificed at sea level. Fetal atrial tissue was harvested for the study. For the control group, pregnant females were reared at sea level for the entirety of gestation and sacrificed near term, comparable to the long-term hypoxic group. The body and organ weights of the long-term hypoxic fetuses did not differ significantly from the normoxic controls. These studies were approved by the IRB and the Animal Care and Use Committee of Loma Linda University, Loma Linda, California under protocol #8110004 on 4 December 2013.

### 4.2. Cell Isolation

Islet-1+ cardiovascular progenitor cell clones were isolated from 5 fetal Suffolk sheep, as previously described by our laboratory [76]. Briefly, atrial cardiac tissue from either normoxic or hypoxic fetal sheep was broken down into 1 mm^3^ sections which were then digested by collagenase (Roche Applied Science, Indianapolis, IN, USA) for 2 h at 37 °C. The cardiac tissue suspension was next filtered through a 40μm cell strainer to isolate CPCs. The resultant cardiovascular progenitor populations were clonally expanded after diluting cells to 0.8 cells per well in a 96-well plate. These monoclonal populations were evaluated for their expression of cardiovascular progenitor cell markers, including Islet-1, c-kit, and SSEA-4, to verify the desired CPC lineage. Cell clones expressing these three markers were used for all of the experiments once monoclonally expanded. 

### 4.3. Hypoxic Cell Culture

The monoclonal populations that were isolated from hypoxic sheep were continuously cultured exclusively under hypoxic conditions when expanded in vitro. Hypoxic CPC clones were maintained in a separate 37 °C incubator under 5% CO_2_ and 1% oxygen. The normoxic/control CPCs arising from normoxic sheep were only cultured under normoxic conditions with 5% CO_2_ and 22% atmospheric oxygen. The culture media and cell expansion procedures remained the same between the normoxic and hypoxic cell clones, and all of the clones were analyzed at low passage.

### 4.4. Quantitative RT-PCR

The normoxic and hypoxic cell lines were trypsinized and stored in RNAProtect (Qiagen, Valencia, CA, USA) until RNA isolation could proceed with the RNeasy Mini Kit (Qiagen, Valencia, CA, USA). RNA quality and quantity were assessed using a Nanodrop 2000 spectrophotometer (Thermo Fischer Scientific, Rochester, NY, USA) and gel electrophoresis. cDNA was prepared using 2µg of RNA and Superscript III, following the manufacturer’s instructions (Life Technologies, Carlsbad, CA, USA). Quantitative real-time polymerase chain reaction (qRT-PCR) was performed using Go-Taq qPCR Mastermix (Promega, Madison, WI, USA) and the iCycler iQ^TM^5 PCR Thermal Cycler (Bio-Rad, Hercules, CA, USA), following a protocol of 94 °C for 10 min and 45 cycles of 94 °C for 15 s, 58 °C for 60 s, and 72 °C for 30 s. RT-PCR products were run on 2% agarose gels with a low mass ladder (Invitrogen, Carlsbad, CA, USA) to confirm the amplification of the appropriate gene. Primers were designed using the National Center for Biotechnology Information Primer-BLAST program and purchased from Integrated DNA Technologies (Coralville, IA, USA). Primer sequences can be found in Table S1.

### 4.5. Western Blot

The cells were detached via a cold trypsin protocol, as previously described by our laboratory [3], and stored in a solution of RIPA buffer, protease inhibitor cocktail, sodium fluoride, sodium orthovanadate, and 0.5M EDTA. This cell solution was agitated for 2 h at 4 °C and subsequently centrifuged at 14,000× *g* and aliquoted for quantification using the Micro BCA Protein Assay Kit (Thermo Fischer, Waltham, MA, USA). These aliquots were run on an automated, gel-free Western blot system (ProteinSimple Wes, San Jose, CA, USA) to quantify specific protein expression across our cell lines. The antibodies used can be found in Table S2.

### 4.6. Flow Cytometry

The CPCs were cultured, trypsinized, and aliquoted for flow cytometry to evaluate the expression of cardiovascular progenitor cell markers including Islet-1, C-kit, CD105, and SSEA-4. The trypsinized cells were washed with 1X PBS (Life Technologies, Grand Island, NY, USA) containing 0.5% BSA (Research Products International Corp, Mount Prospect, IL, USA) and 2mM EDTA (Sigma Aldrich, St. Louis, MO, USA). The treated CPCs were fixed with 4% paraformaldehyde (PFA) (Sigma Aldrich, St. Louis, MO, USA), permeabilized in 0.1% Tween-20 (Sigma Aldrich, St. Louis, MO, USA), blocked in 0.6 M glycine (Sigma Aldrich, St. Louis, MO, USA) solution containing 10% BSA (Research Products International Corp, Mount Prospect, IL, USA), and stained for cardiovascular progenitor cell lineage markers. Once stained, the CPCs were analyzed in a MACSQuant analyzer (Miltenyi Biotec, Auburn, CA, USA). Data quantification was accomplished using FlowJo 10.7 (Ashland, OR, USA). UltraComp eBeads (Life Technologies, Grand Island, NY, USA) were used to compensate, following the manufacturer’s directions.

### 4.7. Cell Cycle

Cells were aliquoted into 250,000 cell lots and stored in 70% ethanol, at −20 °C overnight. These cells were then incubated in RNase A (Fisher Scientific, Pittsburg, PA, USA) for 60 min at 37 °C. Propidium iodide was used to stain the samples prior to cell cycle analysis via a MACSquant analyzer (Miltenyi Biotec, Auburn, CA, USA). The data were analyzed on FlowJo v10.7 (Ashland, OR, USA) using the Dean-Jett-Fox model cell cycle analysis tool.

### 4.8. Transwell Invasion Assay

The upper chambers of Corning HTS Transwell^®^ plates (8.0-µm pore size, Venlo Limburg) were coated with Cultrex^®^ basement membrane extract (Trevigen, Gaithersburg, MD, USA). Fetal cardiovascular progenitor cells were suspended in starvation media composed of 98.5% Iscove’s Modified Dulbecco’s Medium with GlutaMax^TM^ (Life Technologies, Carlsbad, CA, USA), 1.0% insulin-transferrin-selenium (Life Technologies, Carlsbad, CA, USA), and 0.5% fetal bovine serum (Thermo Scientific, Waltham, MA, USA) and plated on the coated wells at 50,000 cells per well. The lower chamber contained cardiovascular progenitor cell growth media with stromal cell-derived factor-1α (SDF-1α, Life Technologies, Carlsbad, CA, USA), a chemoattractant, at a concentration of 100 ng/mL. The cells were incubated for 48 h at 37 °C, dissociated, stained with calcein AM (BD Biosciences, San Jose, CA, USA), and analyzed using a FLx900^TM^ microplate fluorescence reader (BioTek Instruments, Winooski, VT, USA).

### 4.9. Ingenuity Pathway Analysis

We input our genetic regulation data into Ingenuity Pathway Analysis (Qiagen, Valencia, CA, USA) to evaluate and assess potential and known pathway interactions between the targeted survival and dedifferentiation pathways. IPA assembled a network of direct and indirect pathway interactions that we used to validate our proposed mechanism of hypoxic regulation. The networks were generated through the use of IPA (QIAGEN Inc., https://www.qiagenbioinformatics.com/products/ingenuity-pathway-analysis).

### 4.10. Statistical Analysis

We used a two-tailed, paired *t*-test to compare the mean of all normally distributed data and a Wilcoxon matched-pairs signed rank test to compare the mean of all non-normally distributed data. For cell cycle and migration, samples in each group were pooled and a two-tailed, unpaired *t*-test was used for normally distributed data, whereas a Mann-Whitney U test was used to compare the mean of all non-normally distributed data. Prism version 7 was used for all statistical analysis, and all data are represented as the mean ± the standard error of the mean. *p*-Values < 0.05 were considered to be statistically significant.

## 5. Conclusions

The generation of the mitotically stable cell lines of ovine fetal cardiovascular progenitors that are characterized by long-term hypoxia-induced enhancements in stemness has the potential to be of great importance for creating sheep models for human cell/tissue engineering and regenerative medicine. The latter are aimed at cardiovascular therapies and cardiosurgical treatments of a variety of cardioangiopathies [3,4]. In addition, the establishment of permanent cardiovascular stem cell clones that exhibit augmented stemness properties as a result of expressing the genotypic and phenotypic traits related to their tissue-specific genomic, epigenomic and proteomic profiles could also be valuable for the generation of nuclear donor cells for modern assisted reproductive technologies (ARTs) such as cloning sheep and other mammalian species by somatic cell nuclear transfer (SCNT) [77,78,79]. Future work in these areas is needed to address these possibilities.

## Data Availability

The data presented in this study are available upon reasonable request.

## Supplementary Materials

The following are available online at https://www.mdpi.com/article/10.3390/ijms22179382/s1.

## Abbreviations

AKT	Ak Strand Transforming
BCL2BSA	B-Cell Lymphoma 2Bovine Serum Albumin
CCND-1	Cyclin D1
c-Kit	KIT Proto-Oncogene: Receptor Tyrosine Kinase
CPC	Cardiovascular Progenitor Cell
CXCR4	CXC Chemokine Receptor 4
DNA	Deoxyribonucleic Acid
EDTA	Ethylenediamine Tetra-Acetic Acid
ERBB	Epidermal Growth Factor
FAK	Focal Adhesion Kinase
HES1	Hes Family BHLB Transcription Factor 1
HIF-1	Hypoxia-Inducible Factor 1
HIF-2	Hypoxia-Inducible Factor 2
HMOX	Heme Oxygenase
IPA	Ingenuity Pathway Analysis
IRB	Institutional Review Board
ISL-1	Islet-1
MAPK	Mitogen Activated Protein Kinase
MYC	MYC Proto-Oncogene
Nanog	Nanog Homeobox
NFKB	Nuclear Factor Kappa Light Chain Enhance of Activated B Cells
NRG	Neuregulin
OCT4	Octamer-Binding Transcription Factor 4
PBS	Phosphate Buffered Saline
PDK	Pyruvate Dehydrogenase Kinase
PIK3C2B	Phosphatidylinositol-4-Phosphate 3-Kinase C2 Domain Containing Beta Polypeptide
PIK3CA	Phosphatidylinositol-4,5-Bisphosphonate 3-Kinase Catalytic Subunit Alpha
PKCPOU5F1	Protein Kinase CPou Domain, Class 5, Transcription Factor 1
RELA	V-Rel Avian Reticuloendotheliosis Viral Oncogene Homolog A
RIPA	Radioimmunoprecipitation Assay
RNA	Ribonucleic Acid
RT-PCR	Reverse Transcription Polymerase Chain Reaction
SDF-1	Stromal Cell Derived Factor 1
SOD2	Superoxide Dismutase 2
SOX2	SRY-Related HMG Box
SSEA-4	Stage Specific Embryonic Antigen 4
Wnt	Wingless Tyrosine Kinase
Yap1	Yes1 Associated Transcriptional Regulator
