# Supplementary material for: Long-Term Hypoxia Maintains a State of Dedifferentiation and Enhanced Stemness in Fetal Cardiovascular Progenitor Cells
Source: Int J Mol Sci. 2021 Aug 29;22(17):9382. doi: 10.3390/ijms22179382 (PMC8431563; doi:10.3390/ijms22179382)
Supplement: Supplementary file 1 [file ijms-22-09382-s001.zip › Supplemental Figures 1302930.pdf]

Expression Analysis - Hypoxia

Summary | Graphical Summary | Canonical Pathways | Upstream Analysis | Diseases & Functions | Regulator Effects | Networks | Lists | My Pathways | Molecules | Analysis Match

Upstream Regulators | Causal Networks

Add To My Pathway | Add To My List | Display as Network | Activity Plot | Customize Table | Mechanistic Networks

Upstream Regulator	Predicted Activation State	Activation z-score	p-value of overlap	Target Molecules in Dataset
PI3K (complex)	Activated	2.564	4.15E-10	↑BCL2, ↑CCND1, ↑CXCR4, ↑MYC, ↑NANOG, ↑NOTCH1, ↑RELA
NFkB (complex)	Activated	2.602	2.67E-09	↑BCL2, ↑CCND1, ↑CXCR4, ↑MYC, ↑NOTCH1, ↑RELA, ↑SOD2, ↑WNT5A
CCND1	Activated	2.219	5.06E-08	↑BCL2, ↑CCND1, ↑MYC, ↑NANOG, ↑NOTCH1, ↑SOX2
STAT3	Activated	2.925	7.59E-11	↑BCL2, ↑CCND1, ↑CXCR4, ↑MYC, ↑NOTCH1, ↑POU5F1, ↑SOD2, ↑SOX2, ↑WNT5A
IGF1	Activated	2.355	1.47E-06	↑BCL2, ↑CCND1, ↑MAPK1, ↑MYC, ↑RELA, ↑SOX2
VEGFA	Activated	2.213	1.24E-06	↑BCL2, ↑CCND1, ↑CXCR4, ↑NOTCH1, ↑SOD2
JUN	Activated	2.180	1.53E-08	↑CCND1, ↑MYC, ↑NANOG, ↑POU5F1, ↑SOD2, ↑SOX2, ↑WNT5A
POU5F1	Activated	2.607	5.37E-11	↑BCL2, ↑CCND1, ↑MYC, ↑NANOG, ↑POU5F1, ↑SOX2, ↑WNT5A, ↑YAP1
PIK3R1	Activated	2.236	2.17E-09	↑BCL2, ↑CCND1, ↑MYC, ↑PIK3CA, ↑SOD2
FGF2	Activated	2.374	2.85E-10	↑BCL2, ↑CCND1, ↑CXCR4, ↑MYC, ↑NOTCH1, ↑POU5F1, ↑SOX2, ↑WNT5A
RELA	Activated	2.567	3.69E-12	↑BCL2, ↑CCND1, ↑CXCR4, ↑MYC, ↑NANOG, ↑NOTCH1, ↑POU5F1, ↑RELA, ↑SOD2
LIF	Activated	2.201	4.16E-13	↑CCND1, ↑MYC, ↑NANOG, ↑POU5F1, ↑SOD2, ↑SOX2, ↑WNT5A, ↑YAP1
EGF	Activated	2.904	5.18E-11	↑BCL2, ↑CCND1, ↑CXCR4, ↑MAPK1, ↑MYC, ↑NANOG, ↑NOTCH1, ↑POU5F1, ↑SOX2
WNT3A	Activated	2.355	5.24E-08	↑BCL2, ↑CCND1, ↑MYC, ↑NANOG, ↑POU5F1, ↑WNT5A
PTEN	Inhibited	-2.178	7.39E-05	↑BCL2, ↑CCND1, ↑CXCR4, ↑MYC, ↑POU5F1

Supplemental Figure S1. The datasets obtained following long term hypoxia exposure were uploaded into IPA for expression analysis. Predicted activation/inhibition status is shown in the highlighted box.

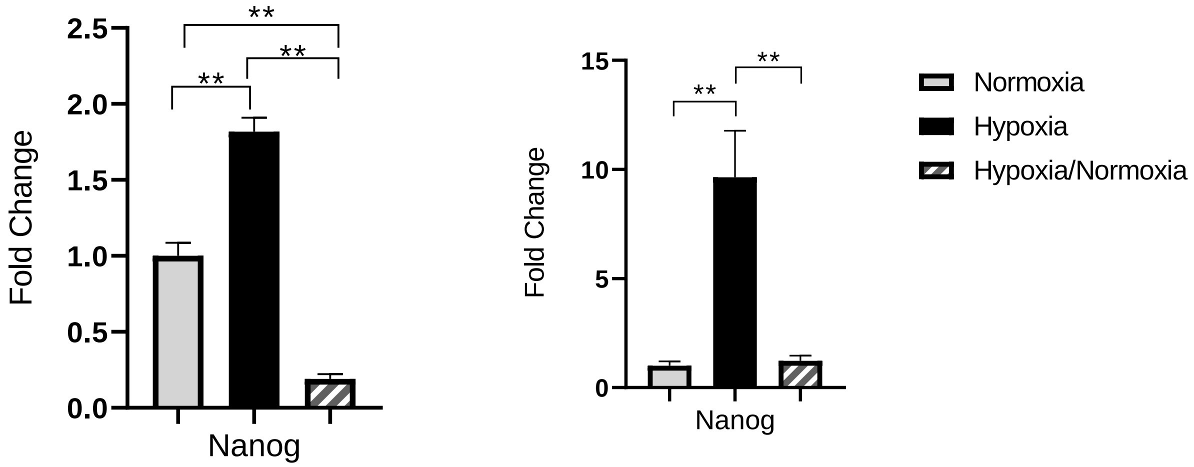

Supplemental Figure S2. Nanog transcripts are significantly elevated when cardiac progenitor cell clones are maintained in long term hypoxic conditions but declines rapidly when the clones previously maintained in hypoxia are subsequently cultured for 72 hours under normoxic conditions as demonstrated by real time PCR. The graphs show two representative hypoxic clones that were returned to normoxic conditions. Four technical replicates were done. Data are reported as the mean ± SEM, ** $p < 0.01$.
