# Supplementary material for: Long-Term Hypoxia Maintains a State of Dedifferentiation and Enhanced Stemness in Fetal Cardiovascular Progenitor Cells
Source: Int J Mol Sci. 2021 Aug 29;22(17):9382. doi: 10.3390/ijms22179382 (PMC8431563; doi:10.3390/ijms22179382)
Supplement: Supplementary file 1 [file ijms-22-09382-s001.zip › Supplementary Table S1 and Table S2 IJMS.pdf]

Supplementary Table S1: Primer pairs for qRT-PCR

**Sheep Genes**	**Forward Sequence**	**Reverse Sequence**
YAP1	TCC CAG ATG AAC GTC ACA GC	TCA TGG CAA AAC GAG GGT CA
HIF1α	GAAGAACTTTTGGGCCGCTC	AGCTGGTGCCAGCAAAGTTA
HIF2 α	ACGCCATCACTGTCTGGTATT	GGTGGGAAGTGACAATGCCT
NRG1	TGG TGA TCG CTG CCA AAA CT	CAGCTGTGACTGGGAGTC G
NRG2	GGT AAC CCT CTG CCC TCC TA	CAT TGC GAA CTG CTG ACA CC
ERBB1	GAC TTT ACT GGG GCC TGA CC	ACG TGT TAC CTG GAA GGC TG
ERBB2	AGA TCC TCA AGG GAG GGG TC	GAA GGT ATA ACG CCC CTC GG
ERRB3	AGT GCC TAT CTT GCC GGA AC	CTT GTA GAT GGG GCC CTT GG
ERBB4	AGT CAC AGG CTA CGT GTT GG	CAG GTT GGA AGG CCA TGG AT
PIK3C2B	CGC AGG TGC CCA GAC A	GTA GAG TGG TTG GAC AGC CC
PIK3CA	AACAATGCCTCCACGACCAT	TCACGGTTGCCTACTGGTTC
PDK3	CCCAGGGACGCTTCCAAATA	CCAAGGCCATAGTTGGTGGT
Wnt3a	TCCTCCATGGTAGAAGCACATC	CTGGGCATGATCTCCACGTA
Wnt5a	CTTCGCCCAGGTTGTAATTGAAGC	CTGCCAAAAACAGAGGTGTTATCC
Wnt9a	GTACCAGTTCCGCTTTGAGC	CTGCCCACTGGGTAAGTCA
Wnt11	GAGGAAGAAAGTCCAGTCCCG	CCGCGAGTCTTGCTAGATGT
JAG	GGAAATCTGCACGGCAGGG	GGATCCCTGGAGCATCACTG
Notch1	CTGCGCGGCCTGATGT	CCCCCTCATGTCTGGTTGTC
Notch2	GATGGCCTGGGTACCTACCGCT	GCAGCGACAACTGTAGCCTCCAA
FAK	CGCAGAGGGGAAGCTTAGTC	ACTCGGCGTGTGATTCAAGT
MAPK1	GGCTGTTCCCAAATGCTGAC	CCCTTGCTAGAGCTCACTGTAT
PKC	GCGAGGGACCATGGCTG	CAAACTTGGCACTGGAAGCC
CCND1	TCGAGCACTTCCTCTCCAAAA	GTTTGCGGATGATCTGCTTGT
MYC	CGCATCAGCACAATTACGCA	ACTCTGGGATCTGGTCACGA
RELA	GCGAGAGGAGCACAGATACC	GGGGTTGTTGTTGGTCTGGA
SOD2	ACCACGCGGCCTACGTGAAC	AGAAAGCCGAGTGTTTCCCTTGGG
OCT4	AGGTGTTCAGCCAAACGACC	TGATCGTTTGCCCTTCTGGC
SOX2	AACTCGGAGATCAGCAAGCG	GGGTGCCCTGCTGAGAATAG
NANOG	TGGGGAATCTTCACCCATGC	AGTTCACCAAACACCCCTGAG
NESTIN	GAATCCCTGAGGGCTCCAGA	CGCTCTTCATTTAGGGACCGT
CXCR4	TTTGGGCAGTTCCTCTGCAA	ACTCACACCCTTGCTGGATG

Supplementary Table S2: Antibodies for Western Blotting and Flow Cytometry

Antibody Western Blot	*Manufacturer*	*Lot*	*Catalog No.*
AKT (pan)	Cell Signaling Technology	20	4691S
Phosphorylated AKT (Ser 473)	Cell Signaling Technology	23	4060S
YAP1	Cell Signaling Technology	2	14074S
Phosphorylated YAP (Ser 127)	Cell Signaling Technology	5	13008T
Actin	Cell Signaling Technology	14	3700S
Antibody for Flow Cytometry	*Manufacturer*	*Lot*	*Catalog No.*
Islet-1	Abcam	GR120150-1	86472
c-kit	Millipore	1995601	FCMAB214P
SSEA4	Biolegend	B153641	330410
CD105	Biolegend	B114774	323205
